# MicroRNA-183 suppresses the vitality, invasion and migration of human osteosarcoma cells by targeting metastasis-associated protein 1

**DOI:** 10.3892/etm.2021.10947

**Published:** 2021-11-04

**Authors:** Xiaoya Sun, Yan Xu, Shanfeng Zhang, Xinjie Li, Yadong Wang, Yan Zhang, Xuefeng Zhao, Yuebai Li, Yisheng Wang

Exp Ther Med 15:5058–5064, 2018; DOI: 10.3892/etm.2018.6068

Subsequently to the publication of the above article, an interested reader drew to the authors’ attention that, on p. 5061, certain of the data panels featured in Fig. 2C (showing the results of Transwell assay experiments) and Fig. 2E (showing the results of scratch-wound healing assay experiments) featured some overlapping data panels, such that the data, which were intended to represent experiments performed under different experimental conditions, may have been derived from the same original source.

The authors have re-examined their raw data and identified the data that should have been included in these two figure parts. The corrected version of Fig. 2 is shown below, now including replacement data for Fig 2C and E. Note that these errors did not have a major impact on either the overall results or on the conclusions reported in this study. The authors regret that these data were mistakenly and inadvertently incorporated into Fig. 2, and apologize to the readership for any inconvenience caused.

## Figures and Tables

**Figure 2:**
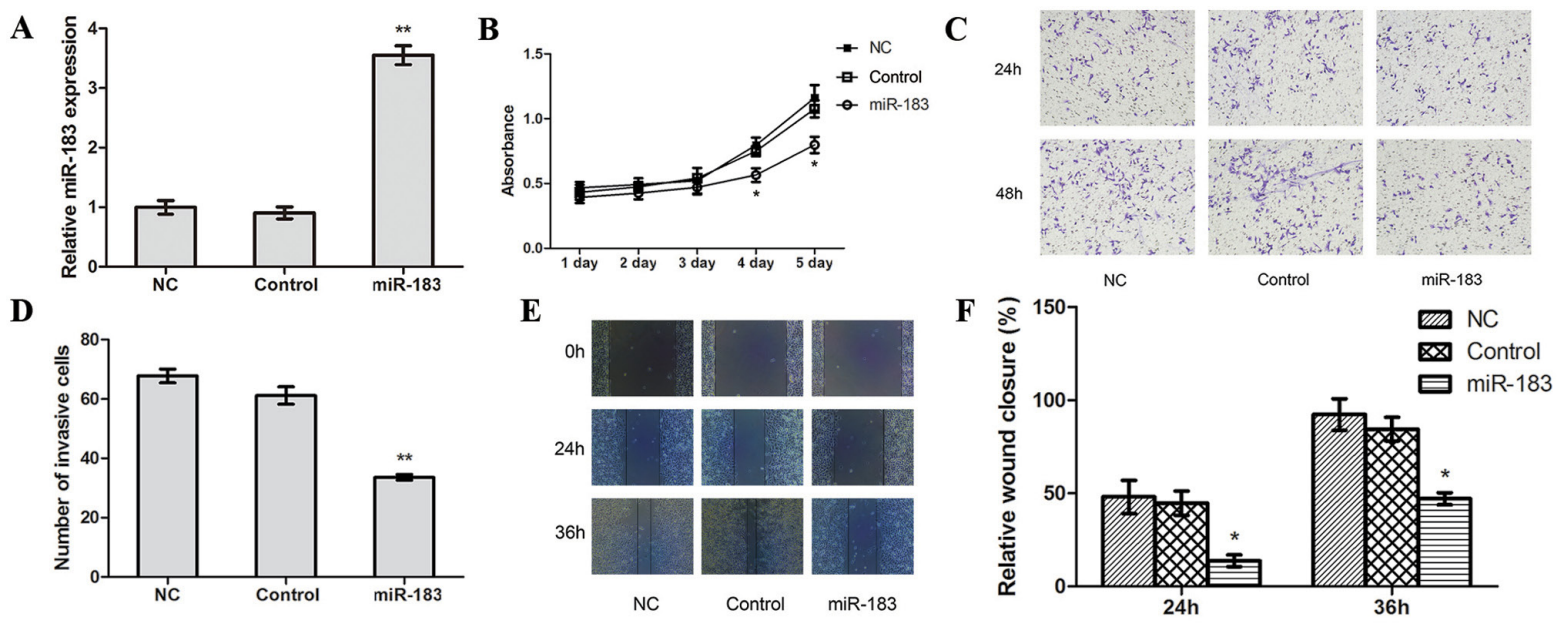
miR-183 upregulation inhibits proliferation, invasion and migration in osteosarcoma cells *in vitro*. (A) The transfection efficiency of miR-183 mimic was assessed using reverse transcription-quantitative polymerase chain reaction. (B) A Cell Counting Kit-8 assay was used to assess proliferation in miR-183 mimic-transfected, control and NC cells. (C) The effect of the miR-183 mimic on cell invasion was detected using a Transwell assay (magnification, x200) and (D) quantified. A scratch-wound healing assay (magnification, x100) was performed, (E) assessed at 0, 24 and 36 h and (F) quantified. ^*^P<0.05 and ^**^P<0.01. miR, microRNA; NC, negative control.

